# Double Feedback Control Method for Determining Early-Age Restrained Creep of Concrete Using a Temperature Stress Testing Machine

**DOI:** 10.3390/ma11071079

**Published:** 2018-06-25

**Authors:** He Zhu, Qingbin Li, Yu Hu, Rui Ma

**Affiliations:** State Key Laboratory of Hydroscience and Engineering, Tsinghua University, Beijing 100084, China; zhuhe14@mails.tsinghua.edu.cn (H.Z.); marui14@mails.tsinghua.edu.cn (R.M.)

**Keywords:** restraint, creep, double feedback method, concrete, temperature stress testing machine (TSTM)

## Abstract

Early-age restrained creep influences the cracking properties of concrete. However, conventional creep measurements require a large number of tests to predict the restrained creep as it is influenced by the combined effects of variable temperature, creep recovery, and varying compression and tension stresses. In this work, a double feedback control method for temperature stress testing was developed to measure the early-age restrained creep of concrete. The results demonstrate that the conventional single feedback control method neglects the effect of restrained elastic deformation, thus providing a larger-than-actual creep measurement. The tests found that the double feedback control method eliminates the influence of restrained elastic deformation. The creep results from the double feedback method match well with results from the single feedback method after compensation for the effects of restrained elastic deformation is accounted for. The difference in restrained creep between the single and double feedback methods is significant for concrete with a low modulus of elasticity but can be neglected in concrete with a high modulus of elasticity. The ratio between creep and free deformation was found to be 40–60% for low, moderate, and high strength concretes alike. The double feedback control method is therefore recommended for determining the restrained creep using a temperature stress testing machine.

## 1. Introduction

The cracking of massive concrete structures due to thermal stresses is a problem that has long been studied by engineers [1]. Dam concrete, a typical type of mass concrete, suffers varying temperature and strong restraint effects during the first days following casting. This influences early-age creep. The early-age creep of concrete can relax more than 50% of the restraint stress [2,3,4]. Creep is one of the most important properties that influence temperature stress in concrete due to relaxation effects [5].

In dam concrete, early-age creep has its own unique characteristics: The temperature in the concrete increases due to the hydration heat and must be controlled below a design value to avoid a large temperature gradient. The temperature stress is not a constant load, instead increasing and decreasing during the hydration process [6,7]. The early-age creep of concrete is also difficult to obtain because both the physical and chemical properties of concrete change simultaneously [4,8]. Therefore, the determination of early-age concrete creep under both varying temperature and restraint conditions is a challenging and a significant topic for research.

The influence factors on the creep, such as temperature, creep recovery, and loading at early-age have been widely studied. Temperature effects have been studied from the perspective of equivalent age [7], hydration degree [9], and transient creep [10,11], among others. Some calculation models, such as the B3 model [12], microprestress–solidification theory [13], rheological model [14,15], and degree of the hydration-based creep model [16,17] have been proposed to predict the effects of temperature on creep. Creep recovery is another important characteristic of creep. Rheological models, such as the modified Double Power Law [7], Kelvin-Voigt [18], and Maxwell [19] can effectively predict the recovery effect. Delsaute [20,21] combined a classical test and a repeated minute–scale–duration loading test to model the recovery effect. 

Loading age can influence the creep magnitude, and the creep loading in early age is more significant than in later age. Existing creep models require modification to predict the extent of this early-age creep. For example, Østergaard [22] suggested a mathematical model for early-age creep by redefining a parameter in the B3 model. Similarly, Wei [23] redefined this parameter in the modified microprestress–solidification model to consider the effects of both temperature and early age on creep. Although the temperature, early age, and unloading factors have been extensively studied, the combined effect of these factors on the very early-age restrained creep of dam concrete is still difficult to accurately predict.

A temperature stress testing machine (TSTM) can be used to study early-age creep under the combined effects of restraint and temperature. The TSTM was developed by many researchers [3,24,25,26,27,28], and a multi-TSTM system controlled by a synchronous closed loop method was constructed by Zhu [29]. The restrained creep has been extracted from free and restrained specimens using a TSTM [25] based on the assumption of linear superposition, which was validated by [7,8,30,31]. In traditional TSTM tests controlled by the single feedback method, the restraint stress is also variable in a compensation cycle, and an additional restraint elastic deformation caused by the varied restraint stress is produced. This varied restraint stress has typically been ignored when decoupling the restrained creep [2,3,4,32].

In order to experimentally investigate the restrained creep of dam concrete under the effects of temperature and restraint conditions at early age, a double feedback control method was developed in this study based on the multi-TSTM system [29]. In the conventional creep test when applying a constant load, a series of tests to consider the varying temperature, alternating tension and compression stress, and different loading ages are required. Based on the multi-TSTM system, the restrained elastic deformation effect was then studied. Finally, a testing method for restrained creep using TSTM is proposed.

## 2. Materials and Methods

### 2.1. Double Feedback Control Method and Creep Calculation Method Using the TSTM

**Figure 1:**
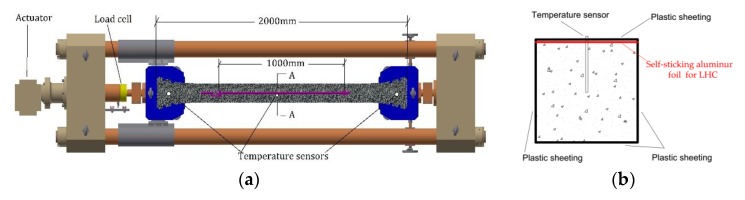
Schematic and the specimen geometry of a temperature stress testing machine (TSTM). (**a**) Schematic of TSTM; (**b**) A-A cross-section view.

In a TSTM as shown in Figure 1a, the specimen deformation is measured directly by a deformation sensor embedded into the concrete and the load is monitored by the load cell. The TSTM can maintain the specimen in a restrained state by continuously checking the specimen deformation. When the preset deformation threshold is exceeded, the actuator of the TSTM is started to compensate for the strain, and the specimen is pushed or pulled to return it to its original length [25]. The number of the compensation cycle is indicated as 1, 2, … *i*, and the time was marked as $t_{1},\;t_{2},\ldots t_{i}$. 

**Figure 2:**
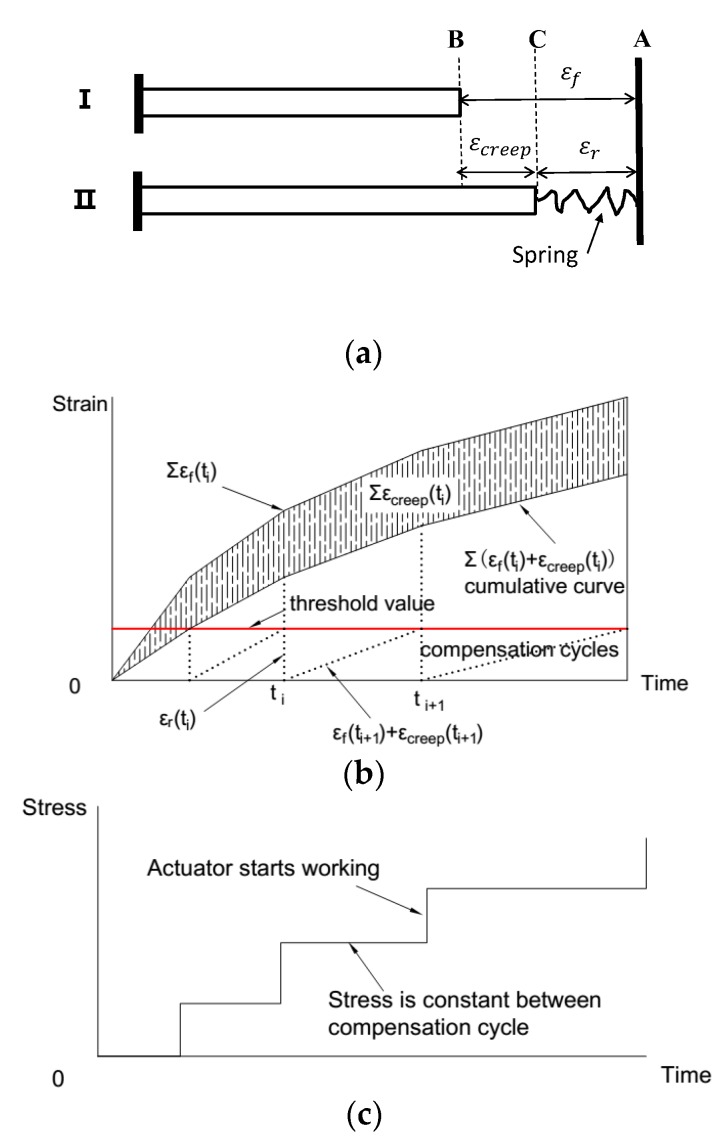
Restrained creep calculation method without considering the restrained elastic deformation, in which $\varepsilon_{r}$ is the strain under the restrained condition, $\varepsilon_{f}$ is the free deformation, and $\varepsilon_{creep}$ is the restrained creep strain. (**a**) Deformation decomposition between the strain compensation cycles; (**b**) Cumulative curve of free deformation, elastic strain and restrained creep; (**c**) Stress history.

In this work, a double feedback control method was developed. Both the load and the deformation of the specimen were monitored and automatically controlled by the computer in real-time. In addition to restoring the specimen to its original length when the deformation reached the preset threshold, the actuator was also working to maintain the load of the specimen as a constant value between the compensation cycles. As shown in Figure 2a, Case I represents the free specimen, and Case II represents the restrained specimen. The cumulative deformation curves are shown in Figure 2b, and the load history of the specimen controlled by double feedback method is shown in Figure 2c.

The deformation can be decomposed into restrained strain and creep as shown in Figure 2a,b, in which $\varepsilon_{f}$, $\varepsilon_{r}$, and $\varepsilon_{creep}$ represent the free strain, restrained strain, and restrained creep, respectively. $\varepsilon_{r}$ is the restrained strain recorded by the strain sensor, and the actuator starts to compensate when $\varepsilon_{r}$ reaches the preset threshold, so the restrained strain is equal to the instantaneous elastic strain induced by the increment load applied by the actuator. The deformations conform to the geometric Equation (1), describing the relationship between the strain compensation cycles, so the restrained creep strain at time $t_{n}$ can be calculated by Equation (2).

(1) $$\;\varepsilon_{creep}\left(t_{i}\right)=\varepsilon_{f}\left(t_{i}\right)-\varepsilon_{r}\left(t_{i}\right)$$

(2) $$\;\varepsilon_{creep}\left(t_{n}\right)=\overset{n}{\underset{i=1}{\sum }}\left[\varepsilon_{f}\left(t_{i}\right)-\varepsilon_{r}\left(t_{i}\right)\right]$$

The restrained creep in the TSTM controlled by the double feedback method can be calculated by Equation (2).

### 2.2. Singles Feedback Control Method and Creep Calculation Method Using the TSTM

**Figure 3:**
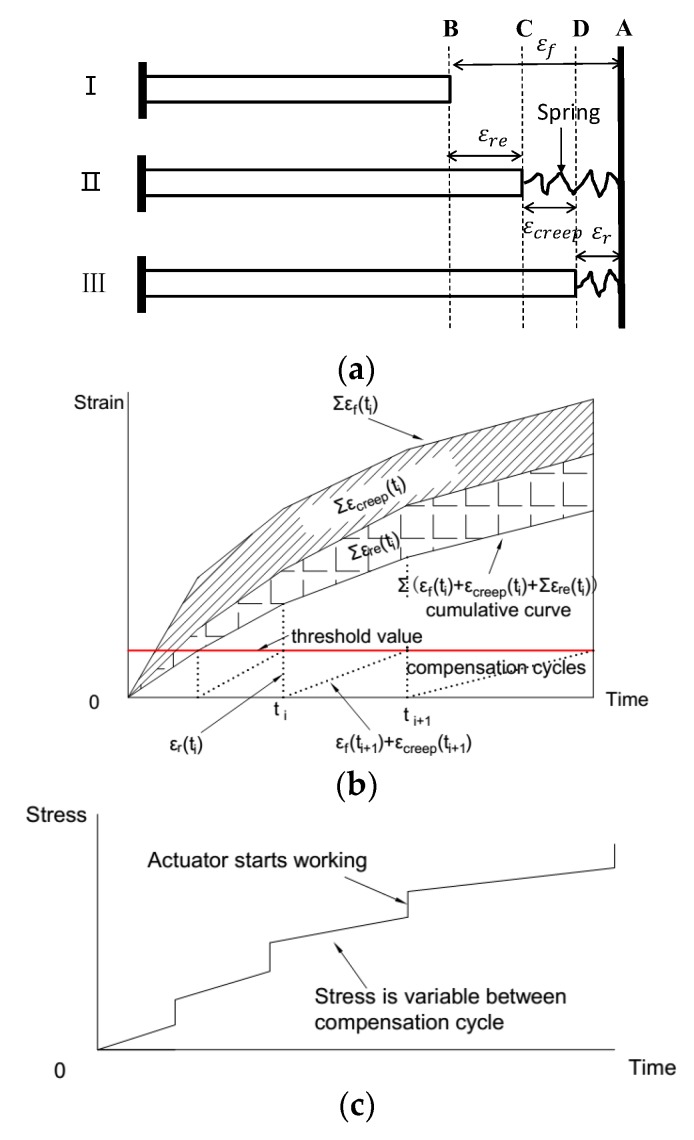
Restrained creep calculation considering the restrained elastic deformation, in which $\varepsilon_{re}$ is the restrained strain. (**a**) Deformation decomposition between the strain compensation cycles of the single feedback control method; (**b**) Cumulative curve of free deformation, restrained elastic deformation, and restrained creep; (**c**) Stress history.

For a traditional TSTM [4,25,33], the control method is called single feedback control method, in which only the deformation is checked to keep the specimen restrained. During the compensation cycle, the load applied to the specimen increases as the deformation increases to the threshold. Both the deformation and the load are variable during the compensation cycle as shown in Figure 3a,c. The difference between the single feedback method and proposed double feedback method is that the load is not a constant value between the strain compensations. The proposed double feedback TSTM is generally conducted as a creep relaxation combining test as both the restrained stress and strain are all variable [33]. An additional restrained elastic deformation, marked as $\varepsilon_{re}$ in Figure 3a,b is produced due to the variable restrained stress during the compensation cycle.

Under the single feedback method [25], the restrained elastic deformation has typically been ignored. The creep in a TSTM test controlled by the traditional single feedback method is also calculated by Equation (2), by decoupling the restrained creep without considering the restrained elastic deformation [2,3,4,32]. In reality, the restrained stress is accordingly variable during a compensation cycle, and an additional restrained elastic deformation will be produced. Consequently, the creep deformation calculated by Equation (2) will be influenced by the restraint stress. 

In Figure 3a, Case I represents the free specimen, Case II represents the restrained specimen under the assumption that the concrete is elastic without considering the creep, and Case III represents the restrained specimen under which concrete is considered to be viscoelastic. Equation (3) can be derived from the force equilibrium equations as follows:

(3) $$\Delta F_{re}\left(t_{i}\right)=E_{ce}\left(t_{i}\right)\times A_{c}\times \varepsilon_{re}\left(t_{i}\right)$$

where $\Delta F_{re}\left(t_{i}\right)$ is the restrained elastic load increment corresponding to the restored elastic deformation increment $\varepsilon_{re}\left(t_{i}\right)$ in compensation cycle i, and can be recorded by the load cell of the TSTM in a compensation cycle; $A_{c}$ is the cross-sectional area of the concrete specimen; $E_{ce}\left(i\right)$ is the concrete elastic modulus, and $\varepsilon_{re}\left(t_{i}\right)$ is the restrained elastic deformation increment of the concrete under the restraint of the device during one adjustment cycle. Based on linear superposition, the deformation relationship is:

(4) $$\varepsilon_{re}\left(t_{i}\right)=\Delta F_{re}\left(t_{i}\right)/\left[A_{c}\times E_{ce}\left(t_{i}\right)\right]$$

(5) $$\;\varepsilon_{f}\left(t_{i}\right)=\varepsilon_{re}\left(t_{i}\right)+\varepsilon_{creep}\left(t_{i}\right)+\varepsilon_{r}\left(t_{i}\right)$$

Therefore, in a complete test period, the creep deformation at time $t_{n}$ can be obtained as shown in Figure 3b and calculated by Equation (6) as follows:

(6) $$\varepsilon_{creep}\left(t_{n}\right)=\overset{n}{\underset{i=1}{\sum }}\left\{\varepsilon_{f}\left(t_{i}\right)-\varepsilon_{r}\left(t_{i}\right)-\Delta F_{re}\left(t_{i}\right)/\left[A_{c}\times E_{ce}\left(t_{i}\right)\right]\right\}$$

### 2.3. Materials

All the raw materials used for mixing the concrete were transported from the construction site of a super-high arch dam. The mix ratios of the concretes are listed in Table 1, in which the water-cement ratio was 0.50. Manufactured sand with a fineness modulus of 2.61 and apparent density of 2790 kg/m^3^ was chosen as the fine aggregate. Limestone gravel with a diameter of 5–20 mm, an apparent density of 2790 kg/m^3^, and a saturated surface dry water absorption rate of 0.21% by mass was employed as the coarse aggregate. The density of the fly ash was 2320 kg/m^3^, and its fineness was 7.6%. The effect of the admixture on the concrete performance is given in Table 2. The constituents of the Portland cement and the fly ash are provided in Table 3. Two kinds of concrete, low-heat cement concrete (LHC) and moderate-heat cement concrete (MHC), were mixed in the same ratios given in Table 1. The only difference between the two mixes was the cement used, detailed in Table 3.

### 2.4. Experimental Procedure

**Figure 4:**
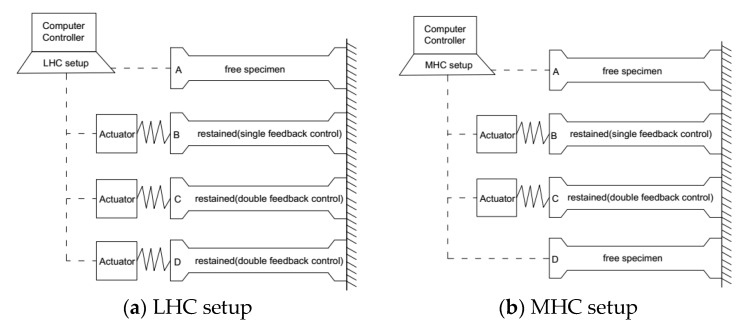
TSTM setups for the testing of low-heat cement concrete (LHC) and moderate-heat cement concrete (MHC) specimens.

Two batches of temperature stress tests were designed for LHC and MHC separately. Four TSTM were employed in each experimental set using a multi-TSTM system [29]. The restraint control methods used by each TSTM are drawn in Figure 4a for LHC and Figure 4b for MHC.

The specimens tested by the TSTM A for LHC and tested by TSTM A and TSTM D for MHC were all set as free ones. The specimens on other TSTMs were set as restrained. Note that the restrained specimens were controlled by one of two methods, single or double feedback. As shown in Figure 4a,b. TSTM B for LHC and MHC were controlled by the single feedback method. Both TSTM C and TSTM D for LHC were controlled with double feedback method to verify the reproducibility of the double feedback method. The MHC-A and MHC-D TSTMs were set with free restraints to verify that the free deformation measured by the TSTMs was representative.

**Figure 5:**
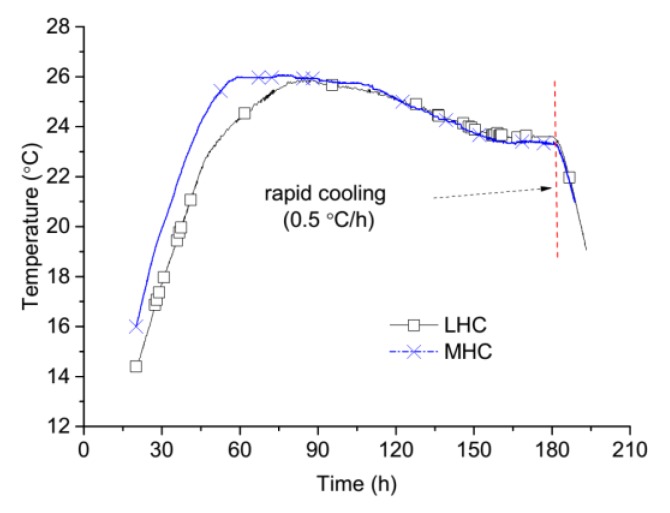
The controlled temperature curves of LHC and MHC measured at a dam construction site.

To simulate a realistic temperature history such as that found at the construction site of a dam, typical temperature history curves measured at the subject site were used as shown in Figure 5. To ensure that the placement temperature was between 14 °C and 16 °C, which was required by the temperature control strategy of the dam construction, all the materials were precooled in an artificial climate laboratory atmosphere of 0 °C for 24 h before mixed. The temperature was reduced at a rate of 0.5 °C/h after the concrete specimens had been cured for seven days.

The restrained test should be initiated as early as possible in order to determine the effects of the early age properties; however, premature failure can occur if the concrete is not of sufficient strength. A variety of methods were studied to determine an appropriate starting time ($t_{0}$ or “zero time”) with respect to aspects of the restrained stress increment [27,28], temperature rate [34], autogenous deformation rate [33,35], and the earliest possible time [3]. The starting time finally selected was approximately equal to the final setting time [36]. In this work, the initial setting time was 14 h and the final setting time was 20 h, so the starting time was chosen as 20 h after the specimens were cured, and at that time the deformation was established as zero. The side formworks were removed 2 h before the starting time to minimize the temperature gradient between the concrete and the surrounding environment.

The concrete was poured into the TSTM and covered by plastic sheets as shown in Figure 1b. After the side formworks were removed, the side surfaces of LHC and MHC specimens were still covered by plastic sheets as shown in Figure 1b. The top surface of the LHC specimens was then sealed with self-sticking aluminum foil to prevent drying, while the top surface of the MHC specimens remained only covered by plastic sheeting in order to conduct a preliminary investigation of drying creep. The detailed experimental procedure of concrete preparation and TSTM protocol can be found in reference [29]. The measured deformation distance of the specimen was 1000 mm, even though the actual length of the specimen was 2000 mm, and a uniform distribution of the restraint stress was obtained between the 1000 mm, of which the schematic of TSTM is drawn in Figure 1a. The deformation sensor has a 0.1 μm resolution and 0.2 μm reproduction accuracy. The concrete temperature was measured by three temperature sensors inserted into the specimen, in the locations shown position can be seen in Figure 1a,b.

## 3. Results and Discussion

### 3.1. Restrained Stress

**Figure 6:**
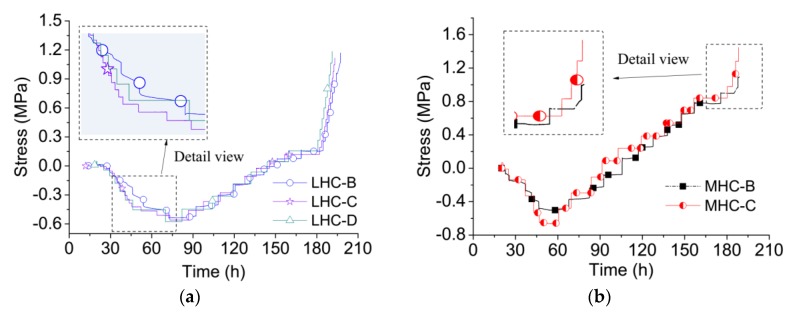
Restrained stress history measured by TSTMs. (**a**) The stress history of LHC; (**b**) The stress history of MHC.

The restrained stress histories of the LHC and MHC specimens are shown in Figure 6. The measured stress difference between LHC-C and LHC-D is insignificant. Both of these TSTMs were controlled by the double feedback method, which indicates that the stresses measured by different TSTMs are reproducible. During the compensation cycles, the stress of specimen LHC-B was varied while that of LHC-C and LHC-D was held constant. Specimens MHC-B and MHC-C manifested the same pattern as the LHC specimen. The results indicate that the restrained stress is variable between the compensation cycles under the single feedback control method. 

Both the restrained stress and strain are variable between compensation cycles, so the temperature stress test is like a kind of creep relaxation combined experiment. The developed double feedback control method holds the load constant, which results in a stepwise creep experimental process. In the temperature increase phase, the compressive stress measured by the single feedback method increased slower than that measured by the double feedback method, as shown in Figure 6a,b During the temperature decrease phase, the tensile stress measured by the single feedback method was smaller than that measured by the double feedback method. Figure 6b shows that the tensile stress of MHC-B was only 1.09 MPa at failure, while specimen MHC-C exhibited a tensile stress of 1.45 MPa at failure. The inset detail view in Figure 6a shows that the stress in LHC-B lags behind that in LHC-C, and the inset detail in Figure 6b shows the same trend for the MHC specimens.

### 3.2. Free and Restrained Deformation

**Figure 7:**
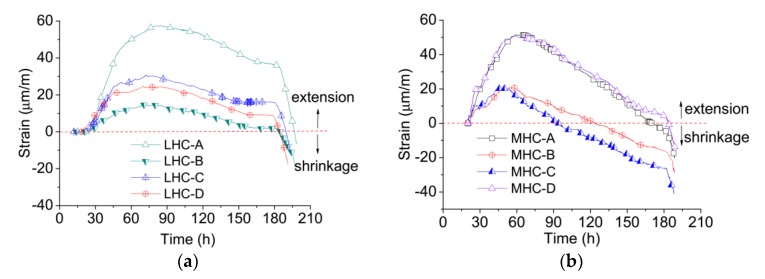
The deformation evolution history from different TSTM tests. (**a**) Deformation results of LHC specimens; (**b**) Deformation results of MHC specimens.

The cumulative deformation results of the LHC and MHC specimen tests are shown in Figure 7a,b, in which positive deformations indicate expansion and negative deformations indicate shrinkage. The value of the free deformation was observed to increase with the temperature and vice versa. In Figure 7a, the maximum free strain is 57.3 με when the temperature is 26.60 °C. The maximum compressive cumulative deformations of LHC-C and LHC-D are only about 30 με because most of the temperature deformation transforms into creep. When the temperature cools from 26.60 °C to 19.50 °C, the free deformation decreases from 57.3 με to 2.2 με and the restrained deformation decreases from 30 με to −9.4 με.

The restrained deformation of LHC-B is smaller than that of LHC-C and LHC-D, corroborating the understanding that the stress determined by the single feedback method lags behind that determined by the double feedback method. An additional restrained elastic deformation is produced in the single feedback method, so the restrained deformation and stress are smaller than those measured in the double feedback method. In the rapid cooling phase, as the restrained stress increased in the single feedback method, the restrained stress is constant in the double feedback method, and an additional restrained stress would retard the increase in the restrained deformation, as shown in Figure 2b. Hence, the change in restrained deformation controlled by the single feedback method is obviously slower than that controlled by the double feedback method.

The free deformations of MHC-A and MHC-D shown in Figure 7b are almost identical, which indicates that the TSTM system provides good deformation measurement and reproducibility. Though the number of specimens is limited in the temperature stress tests, the results indicate that the proposed TSTM system provides high measurement accuracy. The maximum free strain is 49.8 με when the temperature reaches 26.00 °C and decreases to −15.6 με as the temperature cools from 26.00 °C to 21.33 °C. The MHC specimens exhibit larger shrinkage than the LHC specimens because the MHC specimens were only sealed by plastic film rather than self-sticking aluminum foil. The free deformation of the MHC specimens contains temperature deformation, autogenous volume deformation, and dry shrinkage deformation. The restrained deformation of MHC-C develops faster than that of MHC-B, which is the same as the LHC, a result of the different feedback control methods.

### 3.3. Restrained Creep

**Figure 8:**
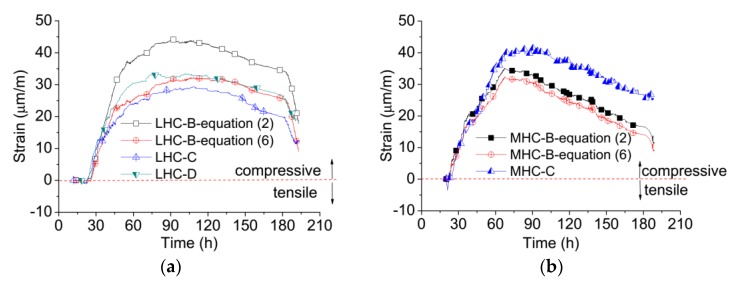
The restrained creep from different TSTM tests. Compressive creep is indicated as a positive value. (**a**) Creep results of LHC; (**b**) Creep results of MHC.

Restrained creep deformations are derived from the free and restrained deformations, with the measurements shown in Figure 8a,b. The creep results of LHC-B, controlled by the single feedback method, are larger than those of LHC-C and LHC-D, controlled by the double feedback method. A restrained elastic stress increment is generated during the compensation cycle, so the creep deformation calculated by Equation (2) includes the restrained elastic deformation, which can be eliminated by Equation (6). The modulus of elasticity (*E*) is a key factor influencing the results, and the *E* values of the LHC and MHC were determined at ages of 1, 3, 5, and 7 days by a standard method [37]. In Reference [37], the *E* values were determined by applying a load of 0.2 MPa/s on the specimen, of which the dimension was 100 × 100 × 300 mm. The *E* value was calculated from the variation in stress amplitude from 0.5 MPa to 40% of the specimen compressive strength at the loading age. The values of *E* provided in Table 4 were calculated based on the average values from three 100 × 100 × 300 mm specimen tests at the given testing age.

The loading stress amplitude affects the determined value of *E*, and the degree of influence of the stress/strength ratio on the *E* value is different at different ages [38]. The stress level and stress increments in the specimens during the temperature stress tests were quite small, so the initial tangent *E*-modulus appeared to be more relevant for use in the stress calculations [33] than those determined from the standard *E*-modulus [37]. The *E* value determined using the lower stress amplitude is larger than that using the high-stress amplitude. However, the *E* values calculated by stress and strain increments from the TSTM yield larger scatter because the increments are so small. The “active method” was then applied [39] by cyclically applying a compressive load corresponding to approximately 10% of the concrete compressive strength. Using this method, the value of *E* looks very similar to the *E* values calculated using the stress and strain increments and is approximately 130% of the *E* values determined by the standard method [37], which are also in accordance with existing research results [40]. Therefore, the *E* values used for calculation in Equation (6) were set to 130% of the values shown in Table 4.

After considering the restrained elastic deformation, the maximum compressive creep of LHC-B can be observed to decrease from 45 με to 30 με as shown in Figure 8a and is almost equal to that of LHC-C and LHC-D. The elimination of elastic strain is clearly necessary for LHC: The creep results from the single feedback method calculated by Equation (6) match well with those of the double feedback method for LHC. The restrained creep of MHC-B is smaller than that of MHC-C, especially after 60 h, as shown in Figure 8b. Because the restrained deformation of MHC-C varies faster than that of MHC-B in Figure 7b, the creep value of MHC-B, determined by Equation (2), is smaller than that of MHC-C. This difference is caused by material dispersion and is not contrary to the behavior of LHC. Because the *E* values of MHC are much larger than those of LHC, the restrained elastic deformation of MHC determined by Equation (5) is smaller than that of LHC. The restrained creep results from MHC-B determined by Equations (2) and (6) show little difference. These results indicate that effects of restrained elastic deformation on restrained creep are more obvious for concrete with a lower *E*. This influence can, thus, be safely neglected for high strength concrete as has been done by some researchers [2,41]. The double feedback method, proposed for determining the restrained creep at early ages, is generally confirmed to be accurate by the creep results of the single feedback method after considering the effects of restrained elastic deformation.

The restrained creep can influence the magnitude of the restrained stress. The restrained stress can be measured by the load cell of the TSTM directly, and the theoretical elastic stress can then be derived by Equation (7).

(7) $$\sigma_{fe}\left(t_{n}\right)=\sum_{i=1}^{n}\Delta \varepsilon_{free}\left(t_{i}\right)\cdot E\left(t_{i}\right)$$

where $\sigma_{fe}\left(t_{n}\right)$ is the theoretical elastic stress at a time $t_{n}$ corresponding to the free deformation, and *E*$\left(t_{i}\right)$ is 130% of the values given in Table 4, according to references [39,40]. The maximum compressive stress in the LHC in Figure 6a is only between 0.54 and 0.58 MPa. However, when the temperature of the concrete increases to its maximum value of 26.60 °C, the maximum free deformation is 57.3 με, corresponding to a theoretical elastic stress of approximately 0.95 MPa. The relaxation is about 40–45% at the maximum temperature. The maximum compressive restrained creep is 25–30 με, and the ratio between the creep and free deformation of LHC is about 43.6–52.4%, which is very close to the relaxation ratio. Similarly, the ratio between the creep and free deformation of MHC is about 50–60%, and the stress relaxation ratio is also about 50–60%. The results indicate that, no matter the material, the restrained creep is 40–60% of the free deformation in the restrained temperature stress test, and the creep-to-free deformation ratio is approximately equal to the stress relaxation degree under the given temperature history. This 40–60% creep-to-free deformation ratio has also been observed in high performance concretes [2,3,41,42] whether or not the temperature histories were constant or variable. Thus, the ratio between creep and free deformation can be used as a measure of stress relaxation [7], and early-age creep could relax 40–60% of the restrained stress at early age.

A TSTM can determine the restrained creep as influenced by the combined factors of temperature and creep recovery from a very early age, which can hardly be obtained using only a test with the conventional creep testing method of applying a constant load [41]. The classical linear viscoelasticity creep theory [43,44,45] can also predict restrained creep, however, the viscoelasticity creep model should consider the combined factors such as variable temperature, alternating tension and compression stress, and the early age effects. A significant amount of testing is required to obtain a creep model that can be used to calculate the restrained creep exactly. Some researchers [46,47] have predicted the restrained stress using a creep model derived from the conventional testing method. However, Kovler [41] found that specific creep values obtained in the plateau from a restrained shrinkage test were smaller than those obtained from a conventional test. Two reasons may account for the difficulty of combining the restrained creep as determined by TSTM with that determined by the conventional method. One reason is that the magnitude of self-induced restrained stress is small. The restrained stress at very early age is near zero, yet the restrained creep is considerable. Therefore, the specific creep as determined by dividing creep strain by restrained stress would result in a large discrepancy. Another reason is that the restrained creep is influenced by many factors, so the classical method, which obtains results by applying only a constant load, is insufficient.

In future work, a creep model considering variable temperature, alternating tension–compression stress, and early age effects will be studied by directly measuring creep and free strains. Furthermore, the restrained creep can be accurately predicted by combining the creep model.

## 4. Conclusions

A double feedback control method using a TSTM was developed based on a multi-TSTM system to measure the early-age restrained creep of LHC and MHC specimens. The conclusions are as follows:

(1) The TSTM is a very useful instrument for determining the early-age restrained creep of dam concrete under the combined effects of varying temperature, creep recovery, tension and compression stress, and early age. The double feedback control method for a TSTM can hold the stress constant during the compensation cycle, eliminating the restrained elastic deformation.
(2) The restrained creep measured by the conventional single feedback TSTM control method neglects the effects of restrained elastic deformation, and as a result, the measured creep is larger than the actual value. A new creep calculation equation for single feedback method was, accordingly, derived. The results calculated with the proposed equation show positive agreement with the results of the more accurate double feedback method.
(3) The difference in restrained creep between single and double feedback methods is significant in low elastic modulus concrete and can be neglected in high elastic modulus concrete.

In summary, the double feedback control method is recommended for determining the restrained creep of concrete using a TSTM. The value of early-age restrained creep is significant and should not be neglected. The ratio between creep and free deformation at an early age may be in the range of 40–60% for LHC, MHC, and high-performance concretes.

## Figures and Tables

**Table 1:** Mix ratios of the concrete specimens used in the experiments (kg/m^3^).

Water	Cement	Fly Ash	Sand	Gravel	Water-Reducing Admixture	Air-Entraining Admixture
130.00	169.00	91.00	727.58	1351.23	1.12	0.074

**Table 2:** Effect of the admixtures on the performance of the concrete.

Admixture	Mix Ratio (%)	Water Reduction Ratio (%)	Air Content (%)	Bleeding Rate (%)	Difference in Setting Time (min)	
					Initial Setting	Final Setting
Water reducing admixture	0.60	19.5	1.9	25	+260	+350
Air entraining admixture	0.008	6.5	5.0	35	+40	+70

**Table 3:** Chemical composition of the materials used (% mass).

Material	CaO	SiO_2_	Al_2_O_3_	Fe_2_O_3_	MgO	SO_3_	R_2_O
Low heat cement	58.7	22.8	4.3	4.3	4.2	3.0	0.3
Moderate heat cement	47.9	25.1	11.3	2.4	5.5	3.0	1.3
Fly ash	3.2	52.4	24.0	9.4	1.1	0.4	0.9

**Table 4:** The modulus of elasticity (*E*) of the low-heat cement concrete (LHC) and moderate-heat cement concrete (MHC) at different ages (GPa).

Concrete		1 Day	3 Days	5 Days	7 Days
LHC	Average value	10.57	14.27	16.83	21.73
	Measured values	11.60, 11.70, 8.40	12.20, 14.40, 16.20	15.30, 16.70, 18.00	19.00, 22.50, 22.00
MHC	Average value	13.90	23.27	24.27	26.67
	Measured values	13.70, 13.80, 14.20	23.70, 23.00, 23.10	24.10, 23.50, 25.22	24.90, 29.50, 25.60
